# Adults with autism are less proficient in identifying biological motion actions portrayed with point‐light displays

**DOI:** 10.1111/jir.12623

**Published:** 2019-04-25

**Authors:** E.‐Y. Hsiung, S. H.‐L. Chien, Y.‐H. Chu, M. W.‐R. Ho

**Affiliations:** ^1^ Graduate Institute of Biomedical Sciences China Medical University Taichung Taiwan; ^2^ Graduate Institute of Neural and Cognitive Sciences China Medical University Taichung Taiwan; ^3^ Department of Physical Therapy China Medical University Taichung Taiwan

**Keywords:** autism spectrum disorder, biological motion, global motion, point‐light display, visual perception

## Abstract

**Background:**

Whether individuals with autism spectrum disorder (ASD) have impairments with biological motion perception has been debated. The present study examined the ability to identify point‐light‐displayed (PLD) human actions in neurotypical (NT) adults and adults with ASD.

**Method:**

Twenty‐seven adults with ASD (mean age = 28.36) and 30 NT adults (mean age = 22.45) were tested. Both groups viewed 10 different biological motion actions contacting an object/tool and 10 without making contact. Each action was presented twice, and participant's naming responses and reaction times were recorded.

**Results:**

The ASD group had a significantly lower total number of correct items (*M* = 29.30 ± 5.08 out of 40) and longer response time (*M* = 4550 ± 1442 ms) than NT group (*M* = 32.77 ± 2.78; *M* = 3556 ± 1148 ms). Both groups were better at naming the actions without objects (ASD group: 17.33 ± 2.30, NT group: 18.67 ± 1.30) than those with objects (ASD group: 11.96 ± 3.57, NT group: 14.10 ± 1.97). Correlation analyses showed that individuals with higher Autism‐spectrum Quotient scale scores tended to make more errors and responded more slowly.

**Conclusion:**

Adults with ASD were able to identify human point‐light display biological motion actions much better than chance; however, they were less proficient compared with NT adults in terms of accuracy and speed, regardless of action type.

## Introduction

Human beings demonstrate remarkable and robust visual sensitivity to the movements of other humans and non‐human agents (Blake & Shiffrar 2007; Pinto & Shiffrar 2009). Even with impoverished visual stimuli such as the point‐light displays (PLDs; Johansson 1973), one can reliably recognise the actions despite substantial noise across space and time (Pinto & Shiffrar 1999), and variations in luminance contrast (Ahlström *et al*. 1997). The information extracted from dynamic PLDs of a moving agent is not limited to the movement *per se*; one can recognise the agent's gender (Pollick *et al*. 2005), emotional state (Pollick *et al*. 2001; Atkinson *et al*. 2004; Chouchourelou *et al*. 2006) and even identity (Loula *et al*. 2005).

Several non‐human species show preferential responses to PLDs depicting the bodily movements of members of their own species. For example, cats can discriminate a point‐light animation depicting biological motion (i.e. a cat walking) from an animation consisting of equivalent local motion vectors without the global configuration (Blake 1993). Dark‐reared newborn chicks preferentially oriented to images of the heads of hens (Johnson & Horn 1988) and the PLDs depicting the biological motion of a hen (Regolin *et al*. 2000; Vallortigara *et al*. 2005). In humans, 2‐day‐old newborns could distinguish between biological and random motion PLDs; they preferred upright over inverted PLDs (Simion *et al*. 2008; Bardi *et al*. 2011). By 14 months of age, infants could tell PLDs depicting disrupted from non‐disrupted interactions between people (Galazka *et al*. 2014). These findings collectively suggest an early emerging mechanism for biological motion perception, a fundamental socio‐cognitive ability to detect and interpret others agents' movements and intentions (Pelphrey & Carter 2008; Kaiser *et al*. 2010b).

Although human beings have an innate propensity to perceive biological motion, it does not exclude the possibility of learning and mastering of such expertise in adulthood. Troje (2008) proposed that biological motion perception shall be regarded as a multi‐level phenomenon including two kinds of distinct contributions, a *local process* that utilises the local motion of the body parts (e.g. limbs) or individual dots and a *global process* that uses form‐from‐motion cues to retrieve the global structure such as the dynamically changing shape of a body in motion. This view is supported by the inversion effect of biological motion perception; both the global/configural information (Bertenthal & Pinto 1994) and local cues such as the direction of motion contribute to the impairment in perceiving PLDs when presented upside down (Troje & Westhoff 2006). Moreover, the visual mechanisms responsible for the local processing may constitute an innate and non‐specific life detection system, whereas an acquired system may be responsible for processing the global shape of more specific identifications of an agent and its actions (Troje & Westhoff 2006; Troje 2008; Chang & Troje 2009).

Growing evidence has shown that individuals with ASD may exhibit aberrant biological motion processing. Neuroimaging studies revealed that brain activity in the superior temporal sulcus, an area associated with biological motion perception (Pyles *et al*. 2007), was reduced in individuals with ASD when viewing PLDs human walkers (Herrington *et al*. 2007; Freitag *et al*. 2008; Kaiser *et al*. 2010b). In non‐clinical populations, individual's neural responses to biological motions were reported to correlate with autistic‐like traits (Puglia & Morris 2017). Individuals' behavioural performance of biological motion task correlates with several traits associated with ASD including social cognition and motor imagery (Miller & Saygin 2013). Moreover, individuals with ASD may exhibit a deficit in global processing and tend to compensate for that with increased involvement of local processing (van Boxtel & Lu 2013; van Boxtel *et al*. 2017).

Developmentally, recent studies showed that children with ASD exhibited less sensitivity to biological motion stimuli comparing with typically developing (TD) peers. For example, young children with ASD showed less spontaneous looking preferences to upright biological motions paired with its scrambled version or a non‐biological object motion (Klin & Jones 2008; Klin *et al*. 2009; Annaz *et al*. 2012; Wang *et al*. 2015). Eight‐ to 10‐year‐old children with ASD did poorly on biological motion tasks portraying human actions but performed normally in a static global form task (Blake *et al*. 2003). Other studies with older children and adolescents proposed a slightly different viewpoint; individuals with ASD were selectively impaired with actions portraying emotional states but not non‐emotional actions (Hubert *et al*. 2007; Parron *et al*. 2008) and performed less efficiently with social interaction displays (Centelles *et al*. 2013). This suggested that individuals with ASD can still recognise bodily movements but have specific difficulties in deducing higher order emotional or social meaning to actions. Two recent studies suggest preserved ability of biological motion perception in individuals with ASD. Cusack, Williams, and Neri (2015) adopted an exclusive set of measurements and concluded that people with ASD interpreted other people's actions adequately and returns functionally intact signals that can be accessed when individuals with ASD are prompted and motivated to do so. Using electroencephalography (EEG) and behavioural measurements, Sotoodeh, Taheri‐Torbati, Sohrabi, and Ghoshuni (2019) showed that children with ASD exhibited the same *mu* suppression as age‐matched controls when viewing PLD and video presentations of human actions.

In Taiwan, the prevalence rate of ASD has increased substantially in the past 15 years, and it is about 0.057% according to a recent national survey conducted in 2016 (The Formosa Autism Foundation 2016). However, research on visual aspects of social cognition such as face and biological motion perception in Taiwanese individuals with ASD is limited (i.e. Chien *et al*. 2014; Wang *et al*. 2015; Lin 2016; Hsiung & Chien 2017). Wang *et al*. (2015) examined biological motion perception in 3‐ to 7‐year‐old Taiwanese children with ASD and age‐matched TD peers with two tasks. In the *looking preference task*, the TD group spontaneously looked longer at the biological motion than the scrambled one while the ASD group had equal preference for both. In the *action identification task* where both groups viewed 12 different PLD clips of human actions, the ASD group made significantly more errors and responded more slowly than did TD children.

Transitioning from childhood into adulthood, people with ASD appear to make improvements in understanding the communicative aspects of other's body movements (Fecteau *et al*. 2003; Hubert *et al*. 2007). It remains unclear whether the aberrant processing of biological motion observed in children with ASD (e.g. Wang *et al*. 2015) persists into adulthood in the Taiwanese population. Thus, the present study investigated biological motion perception in adults with ASD and neurotypical (NT) adults with an *action identification task*. We focused on individuals with ASD without an intellectual disability because the task required a reasonable level of language proficiency. Moreover, we divided the 20 actions into two categories: actions without objects/tools (e.g. running and standing up) and actions involving objects/tools (e.g. playing pool and sweeping floor). We consider that successful identification of the two types of actions must exploit both global and local processing, with the global processing being weighted more for actions involving holding objects. One must utilise a stronger global cue to integrate all visible information and comprehend the actions with the ‘invisible’ objects/tools (Ward *et al*. 2002; Miller & Saygin 2013; van Boxtel & Lu 2013).

We have several hypotheses. First, we expect an action type main effect; the actions involving holding objects would be more difficult to identify for both groups, as these actions require stronger global processing to integrate information about the visible body parts and the ‘non‐visible’ object part. Second, if the impairment observed in childhood persists into adulthood, we expect a group main effect; the mean accuracy in adults with ASD shall be significantly lower than that of the NT adults. Alternatively, the absence of group main effect suggests that individuals with ASD make notable progress in perceiving and understanding other's actions during development. Third, if the impairment is rather severe, we expect a significant action type × group interaction effect; the ASD group may exhibit a larger deficit with the actions involving objects/tools, as these actions may be more difficult to interpret than those actions without objects/tools. We also analysed the correlations between the individuals' performances and their Autism Quotient (AQ) scores.

## Methods

### Participants

Twenty‐seven adults with ASD (15 men, mean age = 28.36, *SD* = 5.46 years) and 30 NT adults (15 men, mean age = 22.45, *SD* = 1.86 years) were recruited. The sample size was calculated according to Rosner (2005) and was determined based on statistical power consideration [i.e. we set the type 1 error (α) to .05, type 2 error (β) to .12, hoping to obtain the power (1‐β) at approximately .88 leading to an expected sample size (N) is approximately 28]. Adults with ASD were recruited via Taiwanese Asperger's club from a private Facebook group; they were primarily from Taipei and Taichung areas. NT adults were mostly undergraduate or graduate students from China Medical University, Taichung, Taiwan. The education level of both groups was well‐matched (ASD group: *M* = 15.56 years, *SD* = 1.69, NT group: *M* = 15.83 years, *SD* = 0.70). Table 1 summarises the group characteristics. Written informed consent was obtained before the experiment. The experimental protocols adhered to the ethical standards of the Helsinki Declaration of 1975. All participants had a normal or corrected‐to‐normal vision (20/20) and self‐reported with no history of visually related problems. Participants in the ASD group were clinically diagnosed as having Asperger's syndrome or autism spectrum disorder by physicians in the government‐appointed hospitals. One additional participant in the ASD group was tested but excluded because of data recording error. Participants received a small cash payment and travelling expense compensation if needed.

**Table 1:** The group characteristics of the ASD and NT participants

	ASD group (*N* = 27)	NT group (*N* = 30)
Gender (men:women)	15:12	15:15
Age (years, *M* ± *SD*)	28.4 ± 5.46	22.4 ± 1.86***
Education (years, *M* ± *SD)*	15.6 ± 1.69	15.8 ± 0.70
AQ score (*M* ± *SD)*	37.6 ± 6.11	18.1 ± 4.50***

The numbers in age, education and AQ score represent the group means (*M*) ± standard deviation (*SD*). AQ, Autism Quotient; ASD, autism spectrum disorder; NT, neurotypical.

*** *P* < .001.

### Apparatus and stimuli

#### Autism‐spectrum Quotient (Chinese version)

We adopted the Chinese version pencil‐and‐paper questionnaire of the AQ (Liu 2008). The questionnaire has 50 questions, assessing five different dimensions: *social skill*, *attention switching*, *attention to detail*, *communication* and *imagination*. Twenty‐five questions are worded to produce an ‘agree’ response if the participant displays an autistic‐like behaviour; the other 25 are worded to produce a ‘disagree’ response if the participant displays an autistic‐like behaviour. The score ranges from 0 to 50; a higher score indicates a higher autistic trait.

#### Biological motion action identification task

**Figure 1:**
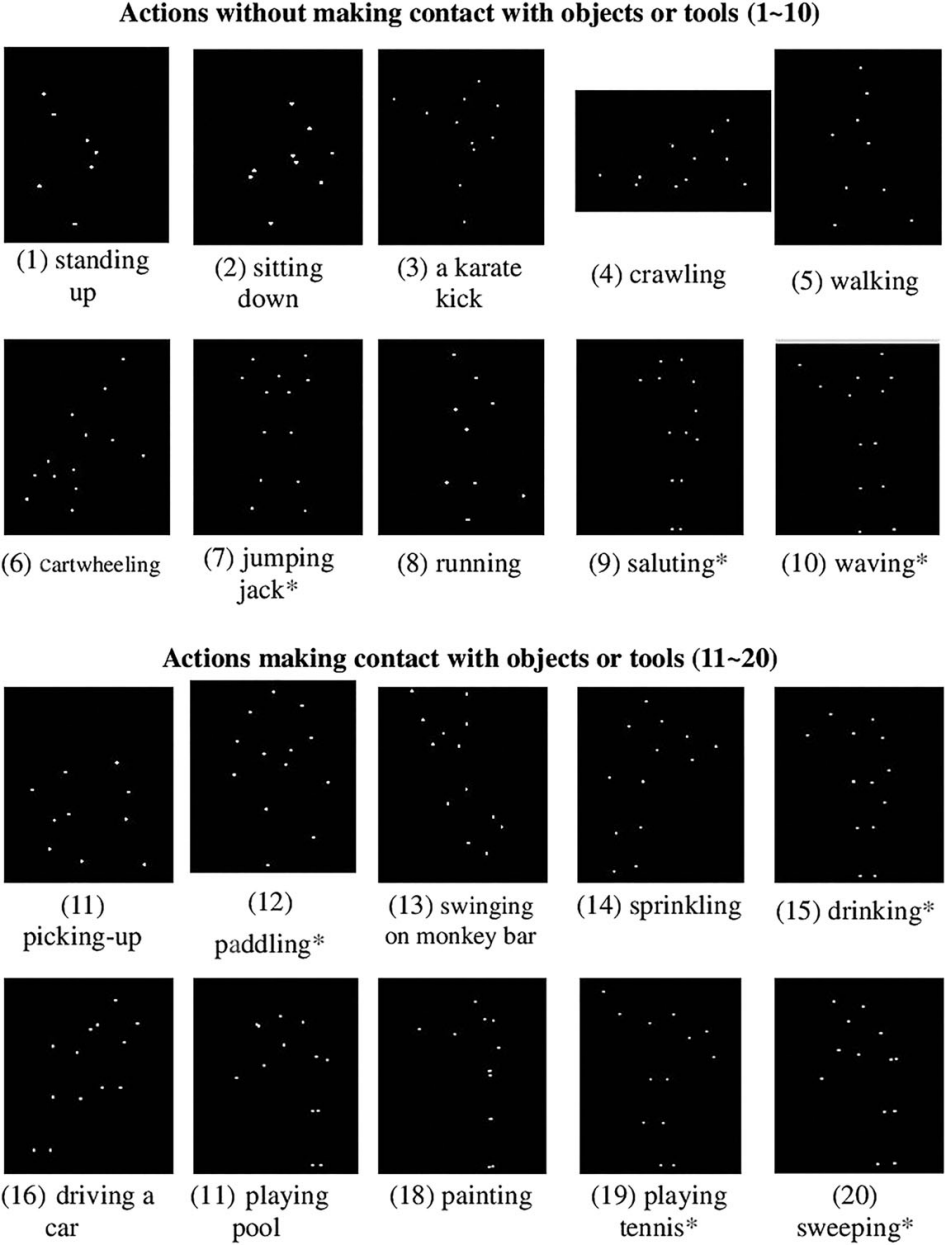
The snapshots of the 20 different human actions depicted in PLDs. Actions (1) to (10) are the ones without objects; Actions (11) to (20) are the ones making contact with objects. *The angle of presentation for jumping‐jack, saluting, waving, paddling, drinking, playing tennis and sweeping is from the ‘front’, and the remainder of the actions is from the ‘side’. PLDs, point‐light displays.

A desktop computer (Acer Veriton M460) with 22″ LCD monitor (Acer P221W, 1440 × 900 pixels, 75‐Hz frame rate) and E‐Prime Professional 2.0 (Psychological Software Tools, Sharpsburg, PA) were used to run the experiment. The stimuli consisted of 20 PLD animations of human actions, selected from two open sources: Spatial Cognition, Action, and Perception Lab at Temple University, Philadelphia, USA (Shipley 2012) and Dr Vanrie & Dr. Verfaillie's Lab of experimental psychology, http://ppw.kuleuven.be/home/English/research/lep/resources/action. The 20 actions consisted of two categories; 10 were the ‘actions without making contact with objects’. This category included *standing up*, *sitting down*, *a karate kick*, *crawling*, *walking*, *cartwheeling*, *jumping‐jack*, *running*, *saluting* and *waving*. The other 10 were ‘actions involving holding objects/tools (but the objects/tools were invisible)’, including *picking‐things‐up*, *paddling*, *swinging on monkey bar*, *sprinkling*, *drinking*, *driving a car*, *playing pool*, *painting*, *playing tennis* and *sweeping*. Figure 1 illustrates the snapshots of the 20 actions. In each trial, an action was played three times with a mean total duration of about 8.7 s (*SD* = 2.3 s). On average, the visual display size of the PLD clips was about 28.3 cm (height) by 17.5 cm (width).

### Procedures

**Figure 2:**
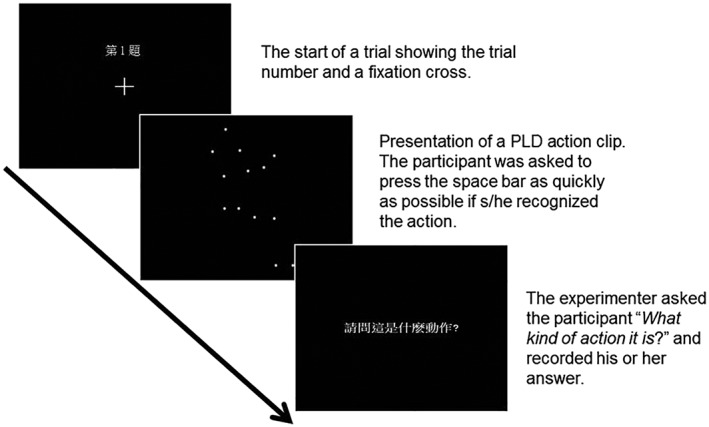
The procedure of a sample trial of the action identification task. PLD, point‐light display.

The study was conducted in a quiet room. The participants filled out the AQ questionnaire first and then performed the computerised *Action Identification Task*. Each participant seated in front of the monitor at a distance of 57 cm, on an adjustable chair allowing his/her eyes to fixate on the centre of the screen. The participants received two practice trials of the same procedure (i.e. bicycling, walking at a 45° angle) before the formal test. Figure 2 illustrates a sample trial. Each trial began with a fixation cross (and a trial number on top of the fixation) for 1 s. The PLD action clip was presented three times for about 7–16 s, and the participant was asked to press the space bar as quickly as possible once they recognised the action. If the participant responded during the PLD presentation, the program would end the PLD clip immediately, recorded the response time, and moved to the next frame prompting ‘what is the action?’ If the participant failed to respond during the presentation, the program recorded the stimulus presentation time as the reaction time. The experimenter then asked the participant ‘what is the action?’ and wrote down his or her answer. A total of 40 trials (20 actions presented twice) were presented in randomised order.

## Results

### Autism Quotient questionnaire scores

**Figure 3:**
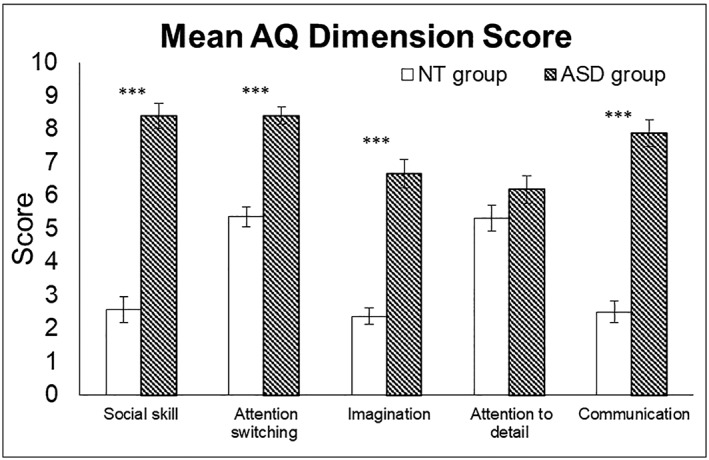
The group mean AQ dimension scores for the NT adults (white bars) and the adults with ASD (patterned bars). The abscissa denotes the five AQ dimensions, while the ordinate denotes the mean scores. Error bars represent the standard errors of the means (^***^ *P* < .001). AQ, Autism Quotient; ASD, autism spectrum disorder; NT, neurotypical.

The mean *AQ* score of the ASD group (*M* = 37.6, *SE* = 1.18) was significantly higher than that of the NT group (*M* = 18.1, *SE* = 0.82), *t*(55) = 13.78, *P* < .001. We further conducted a two‐way mixed analysis of variance (ANOVA) on AQ dimension score with *Group* as the between‐subject factor and *AQ dimension* as the within‐subject factor. The *Group* main effect was significant, *F*_1,55_ = 189.960, *P* < .001, *η* ^2^ = .775, the mean *AQ* dimension score of the AS group (*M* = 7.52, *SE* = 0.21) was significantly higher than that of the NT group (*M* = 3.63, *SE* = 0.19). The main effect of *AQ dimension* was significant, *F*_4,52_ = 14.151, *P* < .001, *η* ^2^ = .205, the mean scores of social skill, attention switching, imagination, attention to detail and communication were 5.49 (*SE* = 0.27), 6.91 (*SE* = 0.20), 4.52 (*SE* = 0.24), 5.76 (*SE* = 0.28) and 5.19 (*SE* = 0.26), respectively. The two‐way interaction effect was also significant, *F*_4,52_ = 18.522, *P* < .001, *η* ^2^ = .252; further analyses comparing the two groups on each dimension revealed significant differences on all but ‘attention to details’ (Fig. 3).

### Biological motion action identification performance

The ‘number of correct responses’ and the ‘speed of identification’ were the two main dependent variables. For each trial, the response was coded as ‘correct’ (1 point) when a participant correctly named or imitated the action using body gestures indicating that they recognised the action. The maximum number of correct responses was 40. The ‘speed of identification’ was the reaction time when the participant pressed the space bar upon recognition. Only the correctly identified trials were included in the ‘speed of identification’.

#### The number of correct responses

**Figure 4:**
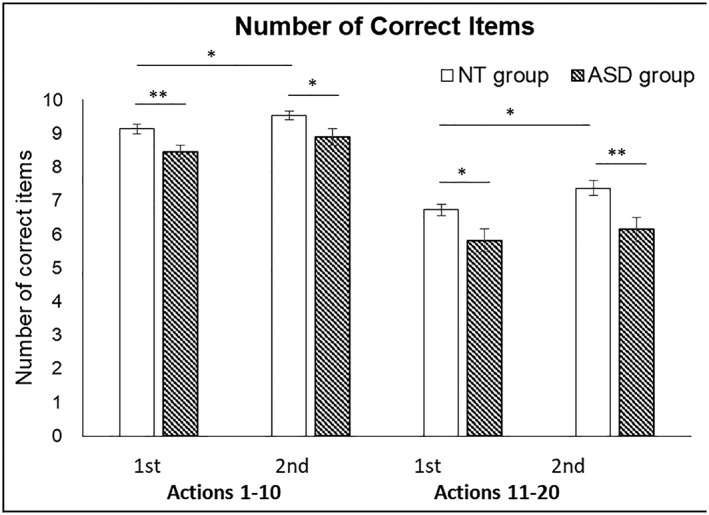
The mean numbers of correct items of the action identification task for the NT adults (white bars) and the adults with ASD (patterned bars). The abscissa denotes the action type in which actions 1 to 10 are actions without making contact to objects and actions 11 to 20 are actions involving holding objects or tools in hand. As each action was presented two times, the data are shown as the numbers of correct items for the first (1st) and the second (2nd) presentation. The ordinate represents the number of correct items. Error bars are the standard errors of the means (^**^ *P* < .01, ^*^ *P* < .05). ASD, autism spectrum disorder; NT, neurotypical.

Figure 4 illustrates the mean numbers of correct responses for both types of actions in both groups. The mean number of total correct items was 32.77 (*SD* = 2.78) in the NT group and 29.30 (*SD* = 5.08) in the ASD group. A three‐way mixed ANOVA was conducted with *Group* as the between‐subject factor, *Action Type* and *Presentation Order* as the within‐subject factors. The *Group* main effect was significant, *F*_1,55_ = 10.517, *P* = .002, *η* ^2^ = .161, the NT group correctly identified more actions (*M* = 8.19, *SE* = 0.18) than the ASD group (*M* = 7.32, *SE* = 0.19). The main effect of *Action Type* was significant, *F*_1,55_ = 211.060, *P* < .001, *η* ^2^ = .793, the actions without making contact to object (*M* = 9.00, *SE* = 0.12) were easier to identify than the actions making contact to objects (*M* = 6.52, *SE* = 0.19). The main effect of *Presentation Order* was also significant, *F*_1,55_ = 11.653, *P* < .001, *η* ^2^ = .415, participants correctly identified more actions for the second presentation (*M* = 7.98, *SE* = 0.15) than the first presentation (*M* = 7.53, *SE* = 0.13). Originally, we had expected an *Action Type* × *Group* interaction effect; however, it was not significant.

We further examined the performance distribution to see if there were certain actions particularly difficult for adults with ASD but easy for NT adults, or vice versa. Table 2 illustrates the frequency counts of correct identification for each of the 20 actions in both groups. As each action presented twice, the maximum number of the count was twice the sample size (*N* = 27 × 2 = 54 for the AS group and *N* = 30 × 2 = 60 for the NT group). The non‐parametric χ^2^‐test of homogeneity was conducted to examine if both groups exhibited a similar distribution across the 20 different actions. The test was not significant, χ^2^ = 0.007, *df* = 19, *P* = .920, meaning that both groups did not differ in the performance distribution of the 20 actions. Namely, the ones that were more likely to be recognised by NT adults were also more likely to be correctly identified by adults with ASD.

**Table 2:** The number of participants in both groups who made a correct response in each of the 20 actions (each presented twice)

	ASD group (*N* = 27) max count = 27 × 2 = 54	NT group (*N* = 30) max count = 30 × 2 = 60
Names of the 20 actions†	Number (counts) of correct responses	Number (counts) of correct responses
(1) Stand up	27	42
(2) Sit down	45	57
(3) Karate kick	47	57
(4) Crawl	54	60
(5) Walk	52	60
(6) Cartwheel	52	59
(7) Jumping‐jack	54	58
(8) Running	52	60
(9) Salute	33	47
(10) Wave	52	60
(11) Paddle	13	20
(12) Swinging on the monkey bar	39	51
(13) Pick‐things‐up	52	60
(14) Sprinkle	12	24
(15) Drink	39	47
(16) Drive	31	44
(17) Play pool	21	33
(18) Paint	43	37
(19) Play tennis	31	51
(20) Sweep	42	56

ASD, autism spectrum disorder; NT, neurotypical.

† Actions (1) to (10) are the actions without objects; actions (11) to (20) are the actions with objects or tools.

#### The speed of identification

**Figure 5:**
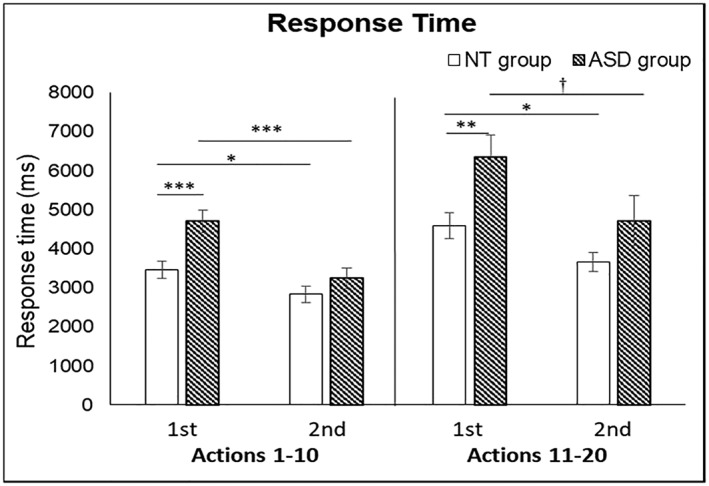
The group mean response times of the correct items for the NT adults (white bars) and the adults with ASD (patterned bars). The abscissa denotes the action type as described in Fig. 4. The data are shown as the mean response times for the first (1st) and the second (2nd) presentation. The ordinate represents the response time (in ms). Error bars are the standard errors of the means (^***^ *P* < .001, ^**^ *P* < .01, ^*^ *P* < .05, ^†^.05 < *P* < .1). ASD, autism spectrum disorder; NT, neurotypical.

Figure 5 illustrates the mean reaction time of the correct responses for both groups. A three‐way mixed ANOVA on the reaction time was conducted with *Group* as the between‐subject factor, *Action Type* and *Presentation Order* as the within‐subject factors. The *Group* main effect was significant, *F*_1,53_ = 7.683, *P* = .008, *η* ^2^ = .127, indicating the NT group (*M* = 3657 ms, *SE* = 250.1 ms) responded significantly faster than the ASD group (*M* = 4685 ms, *SE* = 273.9 ms). The main effect of *Action Type* was significant, *F*_1,53_ = 38.895, *P* < .001, *η* ^2^ = .423; the reaction time for actions without making contact with objects (*M* = 3593 ms, *SE* = 156.1 ms) was shorter than that for the actions involving holding objects (*M* = 4749 ms, *SE* = 248.2 ms). The main effect of *Presentation Order* was also significant, *F*_1,53_ = 51.513, *P* < .001, *η* ^2^ = .493; the reaction time for the first presentation (*M* = 4852 ms, *SE* = 234.0 ms) was significantly longer than that for the second presentation (*M* = 3489 ms, *SE* = 179.1 ms), indicating a practice effect. The two‐way interaction effect of *Presentation Order* × *Group* was significant, *F*_1,53_ = 7.379, *P* = .009, *η* ^2^ = .122; the difference of mean reaction time between the first and second presentation in NT group (*M_first* = 4080 ms, *SE* = 315.5 ms; *M_second* = 3233 ms, *SE* = 241.5 ms) was smaller than that in ASD group (*M_first* = *5624* ms, *SE* = 345.6 ms; *M_second* = 3745 ms, *SE* = 264.5 ms). Other interaction terms were not significant.

### Correlations between Autism Quotient score and the task performance

**Figure 6:**
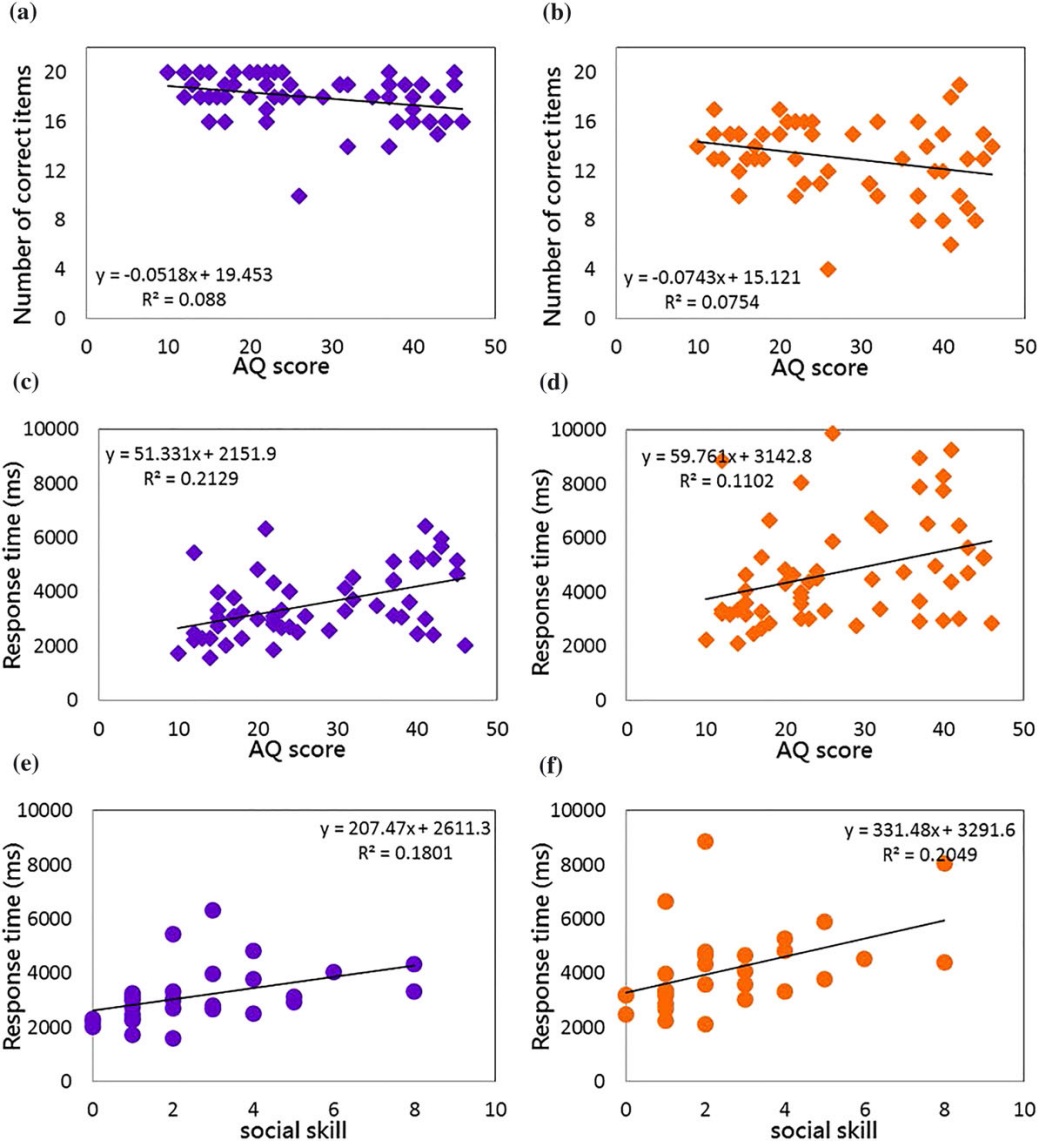
Correlations between individual's AQ score and the biological motion identification performance. Panels a and b: The scatter plots showing the individual's numbers of correct responses in identifying the actions (1–10) without objects (panel a) and those actions (11–20) with objects (panel b) as a function of the AQ scores. The data include both the ASD and the NT groups (total *N* = 57). Panels c and d: The scatter plots of the individual's response time of correct items in identifying the actions (1–10) without objects (panel c) and the actions (11–20) with objects (panel d) as a function of the AQ scores. All participants' data are included except for one (*N* = 56). Panels e and f: The scatter plots of the NT individual's (*N* = 30) response time in identifying the actions without objects (panel e) and the actions with objects (panel f) as a function of the scores on the social skill dimension. The NT participants show a positive correlation between the response time and the social skill score. AQ, Autism Quotient; ASD, autism spectrum disorder; NT, neurotypical. [Colour figure can be viewed at http://wileyonlinelibrary.com]

To reveal whether individual differences in AQ score correlated with the performance of biological motion action identification, we conducted Pearson's bivariate correlations between AQ dimension scores and the number of correct items, the speed of identification for all participants (see Table 3 and Fig. 6 for details).

**Table 3:** Correlations between AQ score and biological motion identification task in all participants (*N* = 57)

	Total AQ score	Social skill	Attention switching	Imagination	Attention to detail	Communication
Number of correct items						
All 20 actions	−.322*	−.350**	−.185	−.235	−.118	−.306*
Actions 1–10	−.297*	−.266*	−.206	−.218	−.164	−.282*
Actions 11–20	−.275*	−.335*	−.135	−.200	−.065	−.261*
Speed of identification						
All 20 actions	.436**	.475**	.213	.451**	.027	.408**
Actions 1–10	.471**	.490**	.284*	.478**	−.015	.468**
Actions 11–20	.329*	.391**	.094	.341*	.067	.284*

AQ, Autism Quotient.

* *P* < .05.

** *P* < .01.

*** *P* < .001.

As expected, significant negative correlations were observed between the individuals' AQ score and the number of correct items of all 20 actions (*r* = −.322, *P* = .015), of the actions without making contact with objects (*r* = −.297, *P* = .025) (Fig. 6a) and of the actions making contact with objects (*r* = −.295, *P* = .039) (Fig. 6b). Likewise, significant positive correlations were observed between the reaction times for all 20 actions (*r* = .436, *P* = .001), for actions without making contact with objects (*r* = .471, *P* < .001) (Fig. 6c) and for actions making contact to objects (*r* = .329, *P* = .013) (Fig. 6d). In other words, individuals with higher AQ scores tended to make more errors and responded more slowly, regardless of action types.

Further analyses on the correlations with the five AQ dimensions reveal some noteworthy associations. For the actions that did not make contact with objects, individuals' number of correct items negatively correlated with the scores on *social skill* (*r* = −.266, *P* = .045) and *communication* (*r* = −.282, *P* = .033) subscales. Individuals' reaction times positively correlated with *social skill* (*r* = .490, *P* < .001), *attention switching* (*r* = .284, *P* = .034), *imagination* (*r* = .478, *P* < .001) and *communication* subscales (*r* = .468, *P* < .001). For the actions making contact with objects, individuals' number of correct items negatively correlated with *social skill* (*r* = −.335, *P* = .011) and *communication* subscales (*r* = −.261, *P* = .050). Individuals' reaction times positively correlated with *social skill* (*r* = .391, *P* = .003), *imagination* (*r* = .341, *P* = .010) and *communication* subscales (*r* = .649, *P* = .034). Moreover, within the NT adults, individuals' scores on the *social skill* subscale positively correlated the reaction times for both types of actions (actions 1–10, *r* = .447, *P* = .013, Fig. 6e) (actions 11–20, *r* = .437, *P* = .016, Fig. 6f), suggesting that NT adults who scored higher on the *social skill* also needed more time to identify the actions regardless of the type.

## Discussions

Using PLDs portraying human actions making contact with or without objects, we examined the performance of action identification (i.e. accuracy and speed of responses) in NT adults and adults with ASD. We obtained several major findings. First, the adults with ASD correctly identified about two‐thirds of the 20 actions; however, they had a lower number of correct items and longer response time for both types of actions compared with NT adults. Our finding that adults with ASD were more erroneous and slower at identifying human PLD actions agreed with several previous studies testing children and young adults (Blake *et al*. 2003; Kaiser *et al*. 2010a; Wang *et al*. 2015).

It is important to note that the adults with ASD in our study had spared capacity of identifying PLD actions; they were simply less accurate and needed more time to process (Moore *et al*.^1997^; Parron *et al*. 2008; Centelles *et al*. 2013; cf. Murphy *et al*. 2009; Cusack *et al*. 2015; Sotoodeh, Taheri‐Torbati, Sohrabi, & Ghoshuni 2019), consistent with Wang *et al*. (2015) showing 3‐ to 7‐year‐old children with ASD made more errors in naming 12 different PLD actions and responded more slowly than TD children. Moreover, the actions that were likely to be correctly named by TD children were also more likely to be correctly named by children with ASD. In our study, the actions that were likely to be correctly identified by NT adults were also likely to be identified by adults with AS (Table 2). Based on the cross‐sectional comparison between the children's data in Wang *et al*. (2015) (p. 70, ASD group: 5.33 correct naming out of 12 actions; TD group: 8.10 out of 12) and the adults' data in the present study (ASD group: 29.3 correct naming out of a total of 40 trials; NT group: 32.8 out of 40), we found an impairment of biological motion perception in adults with ASD but the group difference was smaller in the adult population. In addition, adults in both groups responded better and more quickly when the same actions were presented in the second time (i.e. the main effect of Presentation Order), meaning that both groups benefited from a general effect of practice in the present study.

Our results deviate from the notion that adolescents and adults with ASD were only selectively impaired at labelling the higher order emotional states (Hubert *et al*. 2007; Parron *et al*. 2008), and the studies demonstrated a reserved ability of biological motion perception (Cusack *et al*. 2015; Sotoodeh, Taheri‐Torbati, Sohrabi, & Ghoshuni 2019). In the present study, the ASD group was less accurate at naming the 20 human actions than NT adults, even if the stimuli did not include any PLDs conveying emotional states. Hubert *et al*. (2007) tested adults with ASD to recognise PLDs of a person's actions, subjective states, emotions and objects and concluded that the participants were only selectively impaired at labelling emotional displays. In their report, the ASD participants did show a decline in the accuracy of naming non‐emotional human actions, as opposed to the control group (Hubert *et al*. 2007). It was possible that the difference did not reach statistical significance perhaps because of a small sample size (*N* = 19) and a large variance in the ASD group. Cusack *et al*. (2015) used a comprehensive set of tasks from motion detection to action identification and did not find a significant difference between the NT and adolescences with ASD in any individual task. However, they did observe a subtle deficit of biological motion perception in the ASD group when combing the results of all the tasks. Lastly, Sotoodeh, Taheri‐Torbati, Sohrabi, and Ghoshuni (2019) adopted both EEG and behavioural measurements and reported that children with ASD exhibited the same *mu* suppression EEG signals as the age‐matched controls when viewing human actions. However, in the behavioural task, children with ASD correctly recognised fewer actions and had significantly slower response times than the TD children, which is generally consistent with the present results.

Individuals with ASD often experience less physical activities than NT peers because of their restricted repertoire of interests and lifestyle. Hence, could it be that less proficiency in action recognition is because they have limited physical and visual experiences with these actions and not because of their ASD diagnosis? Indeed, people with ASD often face challenges in physical activities than NT individuals, and it has been reported that individuals with ASD showed impairments in motor skills (Baranek *et al*. 2005; Green *et al*. 2009). However, because we did not directly test motor skills in the present study, we are unsure about whether impairment in motor skills or lack of previous experiences cause the less proficient performance in the ASD group. Perhaps including athletes (i.e. those who have excellent overall motor skills) with people with ASD in the future study would provide an opportunity to disentangle the influence of motor skills and ASD.

Furthermore, could it be that their slow response in action recognition is because they were less motivated to complete the task than the NT adults? We were unable to test the motivation level in the present study. However, based on our observations of the participants in the ASD group as well as their performances, we have reasons to believe that they were highly engaged and motivated to do the task. For example, although the ASD group showed lower accuracy, the experimenter observed that the ASD adults looked carefully at each of the test stimuli and tried hard to recognise the actions throughout the test. At the end of the task, almost every participant in the ASD group wanted a debriefing. They asked the experimenter not only the purpose of the study but also the biological motion actions that they could not recognise – they wanted to know what they have missed indicating a high degree of motivation and interest in the experiment; thus, their performance did not appear to be because of a lack of motivation.

We found that the actions involving holding objects/tools were indeed more difficult to identify than those without making contact with objects/tools. As expected, the NT group performed almost perfect in naming the actions without objects (9.33 out of 10). Among these actions, ‘salute’ and ‘standing up’ were relatively more difficult to identify for the ASD group. For the actions with objects or tools, the reaction time of naming these actions was longer in both groups, reflecting a relatively more complex process as these actions may require stronger global processing to integrate the shape of the body parts and the ‘non‐visible’ object part. Among these actions, the ‘paddling (involving holding an invisible paddle)’, ‘sprinkle (holding an invisible bucket)’ and ‘playing pool (holding an invisible cue)’ were the most difficult ones for both groups. The fact that we did not observe a significant *Action Type* × *Group* interaction indicated that the actions making contact with objects were also difficult for NT adults. It is conceivable that poor recognition could be because of unfamiliarity with these and the need to fill in the critical ‘invisible object or tool’ (Ward *et al*. 2002). Again, because we did not measure previous experience in the present study, we remain unsure about why some actions are harder than others. Future studies should include a checklist or questionnaire surveying a wide range of human actions to get an estimate of the individuals' previous experience.

Finally, collapsed across the two groups, we found that individual AQ scores correlated with biological motion task performance in two ways. The AQ scores correlated negatively with the numbers of correct items and positively with the reaction times, meaning that the individuals with higher scores tended to make more errors and needed more time to identify the actions. Within the NT adults (*N* = 30), the individual scores on the *social skill* subscale correlated positively with the response time, suggesting that the NT adults who also scored high on the *social skill* subscale (i.e. less socially capable) tended to respond more slowly as well. This finding is consistent with some recent studies. For example, Miller and Saygin (2013) used biological motion tasks to explore differences in sensitivity in social/cognitive abilities and motor imagery and showed a significant correlation between the severity of AQ score (Baron‐Cohen *et al*. 2001) and the sensitivity of global form cues. In a similar vein, van Boxtel *et al*. (2017) examined biological motion perception involving different degrees of global processing in healthy population and found that individuals with higher AQ scores tended to perform poorly at recognising meaningful human interactions. These findings provide converging evidence that the global processing of biological motion perception – retrieving the configuration of the acting agent from dynamically changing the shape of a body – seems to be affected in people with a high degree of ASD.

Why would global processing correlate strongly with an individual's social skills? Troje (2008) proposed that biological motion perception involves both global (form‐from‐motion cues) and local (local motion of body parts) processing. Moreover, the visual mechanisms for the local processing may constitute an innate and non‐specific life detection system; whereas an acquired system may be responsible for processing the global shape of specific identifications of an agent and its actions (also see Chang & Troje 2009). Following this line of thinking, it may be that individuals with good social skills have accumulated substantial experiences in interacting with people and observing actions performed by other agents. Those experiences might improve the global processing of biological motion.

## Conclusion, limitation and future work

In summary, the present study delved into an important question about the association between biological motion perception and ASD. Adults with ASD were able but less proficient in recognising PLD human actions regardless of action types. Moreover, adults with ASD needed more time to identify PLD actions; nevertheless, they responded better and faster when the same actions were presented for the second time. These findings are consistent with the Robertson *et al*. (2012) claim that the deficit of global motion perception in individuals with ASD may result from the atypical integration of motion signals during the construction of a global percept. Some limitations of the present study include the age difference between the groups (the ASD group was older). Second, we were unable to assess participants' IQ during the experiment. Also, while the main focus of our study was comparing the performance of action identification between the two groups, in the future, we will adopt multiple tasks and eye‐tracking device to explore the contribution of local versus global processing. Additionally, extending the study to different clinical populations (e.g. adults with personality disorders) and including aspects such as participant's previous physical or visual experiences with the stimuli are potential avenues for future research.

## Conflict of Interest

The authors report no conflict of interests.

## Source of funding

This project was supported in part by Taiwanese Ministry of Science and Technology (MOST) grant numbers 104‐2410‐039‐003‐MY3 and 107‐2632‐B‐039‐001‐MY3 to Dr S. H. L. Chien and by MOST Student's Grant: grant number 104‐2815‐C‐039‐077‐H to E. Y. Hsiung.
